# Hypertension Secondary to Amitriptyline Use as Prophylactic for Migraine in a 26-Year-Old Man

**DOI:** 10.7759/cureus.12848

**Published:** 2021-01-21

**Authors:** Mohammed Hmoud, Faisal Al-Husayni, Abdulaziz Alzahrani, Abdulrahman Alharthi, Emad Alwafi

**Affiliations:** 1 College of Medicine, University of Bisha, Bisha, SAU; 2 Neurology, National Guard Hospital, King Abdulaziz Medical City, Jeddah, SAU; 3 Internal Medicine, National Guard Hospital, King Abdulaziz Medical City, Jeddah, SAU; 4 Internal Medicine, King Abdullah International Medical Research Center, Jeddah, SAU; 5 Family Medicine, Ministry of Health, Alkhobar, SAU; 6 College of Medicine, King Saud Bin Abdulaziz University for Health Sciences, Jeddah, SAU

**Keywords:** amitriptyline, migraine, side effects, hypertension, high blood pressure, headache, adverse effects, secondary hypertension

## Abstract

Amitriptyline is one of the leading medications used for migraine as prophylaxis. Amitriptyline may cause various side effects ranging from mild symptoms such as constipation and dry mouth to severe adverse events such as seizures and coma. Here we present a case of a young gentleman who was started on amitriptyline for migraine but developed hypertension. The patient’s blood pressure normalized after stopping amitriptyline but became elevated when reintroduced. The case highlights the possibility of amitriptyline-induced hypertension even without concomitant medication use or high doses. We recommend regular blood pressure monitoring for patients on amitriptyline regardless of the dose.

## Introduction

Migraine headache is a common type of severe headache associated with symptoms like nausea, aura, and photophobia. Migraine headaches were once believed to be due to alteration of cranial blood vessels' tone, which produces different migraine manifestations [1-3]. Most current evidence supports that a primary neuronal dysfunction and the subsequent intra-cranial and extra-cranial changes are behind the migraine's four known phases. The pathophysiology includes cortical spreading depression, trigeminovascular system activation, and increased sensitization of neurons, among other causes [4,5]. Migraine can be classified as episodic (less than 15 days/month) and chronic migraine (more than 15 days/month).

The worldwide prevalence of migraine is around 10%, with higher rates found in North America, South and Central America, Europe, Asia, and Africa, respectively [6]. In comparison, a study in Saudi Arabia showed migraine prevalence as high as 26%, with female predominance [7].

Acute episodic migraines are treated with non-steroidal anti-inflammatory drugs (NSAIDs) and triptans, while several lines of prophylaxis are offered for chronic migraines. Among the first line is amitriptyline, a tricyclic antidepressant (TCA) proven to be a practical choice to prevent migraines but is less favorable for its side effects profile than other drugs [8,9]. Common side effects include but are not limited to tachycardia, increased appetite, weight gain, urinary retention, dry mouth, blurry vision, and constipation [10,11].

Herein, we describe a case of a young gentleman with migraine who received amitriptyline as prophylaxis but developed hypertension and resolved once stopping the drug.

## Case presentation

A 26-year-old male, who is medically and surgically healthy, presented with a six-month history of right-sided throbbing headache associated with nausea, vomiting, and photophobia nearly every day. The headache is preceded by 15-30 minutes of blurry vision. Initially, the pain was relieved by lying down in a dark, quiet room or oral acetaminophen. Later, the patient required ibuprofen to alleviate the pain as the headache would persist for two days. Initial examination showed a blood pressure of 123/69 mmHg, a heart rate of 72 beats per minute, and a normal respiratory rate. The neurological examination was completely unremarkable. The basic blood tests were within normal limits. 

The patient was diagnosed with migraine headache and started on flunarizine 10 mg once daily. A month later, the patient did not feel any improvement; thus, he has switched to amitriptyline 10 mg once daily. After a month, the dose was increased to 25 mg as there was no improvement. The patient migraine attacks improved to once monthly; however, the patient noticed a new headache character affecting the head's posterior part, not similar to his previous migraine three months after starting amitriptyline. The examination was normal, apart from a blood pressure of 161/82 mmHg. He was asked to monitor his blood pressure at home, which showed an average reading of 160/80 mmHg with few readings reaching 180/90 mmHg. He denied any history of other medications or illicit drug use.

The patient decided to stop amitriptyline and monitor his blood pressure, which normalized after one week. On the next clinic visit, his blood pressure was 111/62 mmHg, and the migraine headache was persistent but responding to analgesics. He was asked to resume amitriptyline to 10 mg and monitor blood pressure, then increase the dose to 25 mg if migraine was not controlled and blood pressure did not increase. After one month, the patient's blood pressure was normal, but he still complains of migraines, so he increased the dose to 25 mg as instructed. However, a week later, the patient's blood pressure readings were high, so the patient stopped amitriptyline. Table 1 shows the patient's blood pressure readings. After six months, the patient was not on any medications, and his blood pressure remained normal. His migraine attacks were much less frequent and responding to simple analgesics.

**Table 1:** Patient’s blood pressure readings before, during, and after amitriptyline use. *On amitriptyline 25 mg. **On amitriptyline 10 mg.

Time	Situation when blood pressure was measured	Blood pressure readings (mmHg)
Day 1	First presentation to the clinic	123/69
After 8 weeks	Second visit to the clinic*	161/82
After 8 weeks	Home reading at 9 AM*	156/77
After 8 weeks	Home reading at 9 PM*	166/84
After 9 weeks	Home reading at 9 AM*	161/89
After 9 weeks	Home reading at 9 PM*	182/99
After 11 weeks	Home reading at 9 AM	119/58
After 11 weeks	Home reading at 9 PM	108/63
After 12 weeks	Second visit to the clinic	111/62
After 15 weeks	Home reading at 9 AM**	113/72
After 15 weeks	Home reading at 9 PM**	99/63
After 17 weeks	Home reading at 9 AM*	143/88
After 17 weeks	Home reading at 9 PM*	149/74
After 24 weeks	Third visit to the clinic	116/65

## Discussion

Amitriptyline, a well known tricyclic antidepressant, can be used in neurology as a prophylactic treatment to reduce migraine attacks' severity and frequency. In migraine, the required dosages are significantly lower in comparison to therapeutic dosages to treat depression. Neurologists prefer amitriptyline if the patient had concomitant sleep disturbance, depression, and anxiety with migraines [11]. Toxicity is generally not evident if taken alone at lower doses without using other centrally-acting medications like anti-epileptic drugs or serotonergic drugs [12].

In general, amitriptyline side effects include weight gain, generalized weakness and sleepiness, alopecia, blurred vision, dry mouth, vomiting, constipation or diarrhea, urinary retention, and can cause serious side effects like arrhythmia, blood dyscrasias, and hypotension [9-13]. The previously mentioned side effects are caused by the amitriptyline mechanism of action, which are serotoninergic, anticholinergic action, α adrenergic blockade, and nor-epinephrine inhibition reuptake at nerve terminals [12,13].

Hypertension, as a complication, is a rare phenomenon. It has been reported in patients taking amitriptyline in toxic levels with or without other medications [14,15]. In the literature, the use of amitriptyline was reported to cause malignant hypertension, corrected by discontinuing amitriptyline and introducing blood pressure medication urgently [16,17]. It is proposed that blockade of norepinephrine reuptake and increased vascular reactivity can result in increased systemic hypertension. The theory, mentioned earlier, was supported by the evidence that the tricyclic antidepressants worsen blood pressure control in patients with pheochromocytoma [13].

Neurological adverse effects of amitriptyline range from mild to severe and lethal. Usually, the drug intoxication neurologically results in confusion, delirium initially, followed by convulsions and coma [16,17]. Notably, stroke-like presentation and autonomic dysreflexia were reported in a patient with spinal cord injury when on amitriptyline-duloxetine combination therapy [14]. The mechanism by which amitriptyline was thought to cause a similar presentation to stroke was theorized to be related to significant vasoconstriction that can lead to ischemic and subsequent infarction [15,18].

There is an apparent temporal relationship between starting amitriptyline and high blood pressure readings in our patient presentation. Evidence of resolution of hypertension after the second time of discontinuing the medication and long-term monitoring supports the same relationship. It is important to note that this case shows that serious side effects can happen even at a low dose without concomitant use of other medications predisposing to hypertensive crises. Fortunately, the management of our case required only outpatient evaluation with only stopping the medication. Serious side effects and intoxication would require urgent evaluation, which may necessitate critical care interventions from airway protection, support, decontamination, hemodynamic stability, and management of seizures [19,20]. Case prognostication is dependent on different factors, including the dose taken, the level of intoxication, the patient's presentation if the central nervous system is involved or not, and response to treatment as well [18-20].

## Conclusions

This case represents the probability of amitriptyline to cause hypertension, even if no other medications were used simultaneously. Regular follow-up for blood pressure, especially on the early days of starting amitriptyline, is considered reasonable, even with small doses. Discontinuing amitriptyline may result in resolving hypertension without the need for antihypertensive medications.

## References

[1] Charles A (2009). Advances in the basic and clinical science of migraine. Ann Neurol.

[2] Charles A (2013). Vasodilation out of the picture as a cause of migraine headache. Lancet Neurol.

[3] Amin F, Asghar M, Hougaard A (2013). Magnetic resonance angiography of intracranial and extracranial arteries in patients with spontaneous migraine without aura: a cross-sectional study. Lancet Neurol.

[4] Cutrer FM (2006). Pathophysiology of migraine. Semin Neurol.

[5] Ashina M (2020). Migraine. N Engl J Med.

[6] Sun-Edelstein C, Mauskop A (2009). Role of magnesium in the pathogenesis and treatment of migraine. Expert Rev Neurother.

[7] Muayqil T, Al-Jafen B, Al-Saaran Z (2018). Migraine and headache prevalence and associated comorbidities in a large Saudi sample. European Neurol.

[8] Silberstein SD, Goadsby PJ (2002). Migraine preventative therapy. Cephalalgia.

[9] Güloglu C, Orak M, Ustündag M, Altunci YA (2011). Analysis of amitriptyline overdose in emergency medicine. Emerg Med J.

[10] Trindade E, Menon D, Topfer LA, Coloma C (1998). Adverse effects associated with selective serotonin reuptake inhibitors and tricyclic antidepressants: a meta-analysis. CMAJ.

[11] Nishimura T, Maruguchi H, Nakao A, Nakayama S (2017). Unusual complications from amitriptyline intoxication. BMJ Case Rep.

[12] (2004). Neurology in Clinical Practice: Principles of Diagnosis and Management. Taylor & Francis.

[13] Dunn FG (1982). Malignant hypertension associated with use of amitriptyline hydrochloride. South Med J.

[14] Parke SC, Reyes MR (2019). Autonomic dysreflexia as a potential adverse effect of duloxetine and amitriptyline combination therapy: a case report. PM R.

[15] Birns J, Henderson K, Bhalla A (2013). Recreational amitriptyline toxicity mimicking basilar artery stroke. Eur J Emerg Med.

[16] Levine M, Brooks DE, Franken A, Graham R (2012). Delayed-onset seizure and cardiac arrest after amitriptyline overdose, treated with intravenous lipid emulsion therapy. Pediatrics.

[17] Roberge RJ, Krenzelok EP (2001). Prolonged coma and loss of brainstem reflexes following amitriptyline overdose. Vet Hum Toxicol.

[18] Pawar PS, Woo DA (2010). Extrapyramidal symptoms with concomitant use of amitriptyline and amiodarone in an elderly patient. Am J Geriatr Pharmacother.

[19] Tatli O, Karaca Y, Gunaydin M, Yurtsever S, Tuten G (2013). Cerebellitis developing after tricyclic antidepressant poisoning. Am J Emerg Med.

[20] Body R, Bartram T, Azam F, Mackway-Jones K (2011). Guidelines in Emergency Medicine Network (GEMNet): guideline for the management of tricyclic antidepressant overdose. Emerg Med J.

